# Role of Genetic Polymorphisms of Deoxycytidine Kinase and Cytidine Deaminase to Predict Risk of Death in Children with Acute Myeloid Leukemia

**DOI:** 10.1155/2015/309491

**Published:** 2015-05-18

**Authors:** Aurora Medina-Sanson, Arturo Ramírez-Pacheco, Silvia Selene Moreno-Guerrero, Elisa María Dorantes-Acosta, Metzeri Sánchez-Preza, Alfonso Reyes-López

**Affiliations:** ^1^Hospital Infantil de México Federico Gómez, Department of Hematology and Oncology, Calle Doctor Márquez 162, Colonia Doctores, Delegación Cuauhtémoc, 06720 Mexico City, DF, Mexico; ^2^Hospital Infantil de México Federico Gómez, Research Division, Calle Doctor Márquez 162, Colonia Doctores, Delegación Cuauhtémoc, 06720 Mexico City, DF, Mexico

## Abstract

Cytarabine is one of the most effective antineoplastic agents among those used for the treatment of acute myeloid leukemia. However, some patients develop resistance and/or severe side effects to the drug, which may interfere with the efficacy of the treatment. The polymorphisms of some Ara-C metabolizing enzymes seem to affect outcome and toxicity in AML patients receiving cytarabine. We conducted this study in a cohort of Mexican pediatric patients with AML to investigate whether the polymorphisms of the deoxycytidine kinase and cytidine deaminase enzymes are implicated in clinical response and toxicity. Bone marrow and/or peripheral blood samples obtained at diagnosis from 27 previously untreated pediatric patients with *de novo* AML were processed for genotyping and *in vitro* chemosensitivity assay, and we analyzed the impact of genotypes and *in vitro* sensitivity on disease outcome and toxicity. In the multivariate Cox regression analysis, we found that age at diagnosis, wild-type genotype of the CDA A79C polymorphism, and wild-type genotype of the dCK C360G polymorphism were the most significant prognostic factors for predicting the risk of death.

## 1. Introduction

Acute myeloid leukemia (AML) is a heterogeneous group of leukemias that result from the clonal transformation of a primitive stem/progenitor cell by more than one genetic aberration. It accounts for 20–25% of all childhood acute leukemias and is responsible for more than one-half of the leukemia deaths.

Current pediatric AML protocols result in 85%–90% complete remission rates [1], with long-term survival rates for patients who achieve remission in the range of 60%–70% and event-free survival rates between 45% and 55% [2–6].

This has made possible thanks to the intensification of chemotherapy, better risk group stratification, increased use of allogeneic hematopoietic stem-cell transplantation, and improvements in supportive care. However, resistance to chemotherapy remains a major cause of treatment failure among pediatric patients with AML, with adverse side effects contributing to morbidity and mortality.

Cytarabine (1-*β*-D-arabinofuranosylcytosine, Ara-C) is a structural analog of deoxycytidine (dCyd) and has been the mainstay of treatment for AML for more than four decades [7]. It is a hydrophilic molecule that requires facilitated diffusion via nucleoside-specific membrane transport carriers [8]. To enter the cell, Ara-C binds to the human equilibrative nucleoside transporter (hENT1), but, when administered at high doses, the drug can be taken up by passive diffusion [9]. Inside the cell, Ara-C is phosphorylated to monophosphate (Ara-CMP) by deoxycytidine kinase (dCK); afterwards, two further phosphorylations are catalyzed by pyrimidine kinases to convert Ara-CMP into the active metabolite ara-cytidine-5′-triphosphate (Ara-CTP) [10]. Ara-CTP competes with dCTP for DNA polymerases *α*, *β*, and *δ*; its incorporation into DNA results in chain termination, with consequent inhibition of DNA and RNA synthesis, causing leukemic cell death. Thus, the intracellular concentration of Ara-CTP is one of the determinants of the antileukemic effect of Ara-C. [11]. The catabolism of Ara-C results from rapid irreversible deamination by cytidine deaminase (CDA) to the noncytotoxic metabolite arabinoside uridine (Ara-U), while the enzyme 5′-nucleotidase (NT5C2) dephosphorylates Ara-CMP, thereby stopping the production of Ara-CTP [12]. Ara-CMP can also be converted into Ara-U by the enzyme deoxycytidylate deaminase (DCTD).

Resistance to Ara-C has been associated with conditions that reduce the intracellular concentration of Ara-CTP, such as inefficient cellular uptake of Ara-C, reduced Ara-C activation, or increased Ara-C inactivation, mainly due to variations in the activity or expression of hENT1 or of the enzymes that participate in the Ara-C metabolic pathway [13]. Malignant cells with a larger intracellular pool of cytosine deoxynucleotides can also be more resistant to Ara-C as a result of a competitive inhibition of dCK activity by dCTP [14].

Several studies have demonstrated genetic variation in key genes of the pharmacokinetic pathway of Ara-C with a consequent variation in the intracellular concentrations of Ara-CTP that can result in differences in toxicity and in the clinical response to AML treatment. These findings provide the molecular basis for explaining interpatient variability to cytidine analogues [15–17]. However, few studies have focused on the association of these genetic polymorphisms with clinical outcome and toxicity in patients with AML treated with cytarabine-based chemotherapy [18–20], and no study has been done on a Mexican population.

We conducted this study in a cohort of Mexican pediatric patients with AML to investigate whether the CDA Single Nucleotide Polymorphism (SNP) A79C and the dCK SNPs C360G and C201T are implicated in clinical response and toxicity of this leukemia.

## 2. Patients and Methods

### 2.1. Patient Samples

Bone marrow and/or peripheral blood samples were obtained at diagnosis from 27 previously untreated pediatric patients with* de novo *AML. We excluded patients with Down Syndrome, promyelocytic leukemia, treatment-related AML, those who abandoned therapy before remission could be documented, and cases without sufficient biomaterial for* in vitro* cytotoxicity assay and genetic analyses.

The diagnosis of AML was based on bone marrow examination, according to the FAB and WHO classification criteria; immunophenotype was determined by flow cytometry, and molecular analyses were performed by real time PCR.

### 2.2. Treatment Protocol

The patients were treated according to a modified NOPHO-AML 93 protocol [3]. All received a first course of Ara-C, etoposide, doxorubicin, and 6-mercaptopurine (6-MP) (ATEDox); since there is no 6-tioguanine available in Mexico, it was substituted by 6-MP. Bone marrow was assessed by morphology on day 16. Good responders received a second ATEDox course, while poor responders received an Ara-C plus mitoxantrone (AM) course. Patients with more than 5% blasts in the bone marrow after the AM course were given a course of high dose of Ara-C plus etoposide (HA2E). If remission was not achieved, the child was classified as a nonresponder. All patients in remission were treated with courses of high-dose cytarabine-based consolidation. The total number of courses was six, with the exception of the patients who required three induction courses to achieve remission. No patient underwent stem-cell transplantation in the first remission.

The patients were stratified according to their response to the first induction course. Good risk patients were those with less than 5% blasts after the first cycle.

Toxicities were assessed using National Cancer Institute common terminology criteria (version 3.0).

### 2.3. Chemicals

Ara-C was obtained from Pfizer Laboratories (Cytosar U), MTT [3-(4,5-dimethylthiazol)-2,5-diphenyltetrazolium bromide] from SIGMA-Aldrich (St. Louis, USA). Tissue culture reagents and supplies were purchased from assorted vendors. PCR primers were obtained from Operon, Inc. (The Woodlands, TX, USA). Taq polymerase and restriction enzymes were purchased from New England Biolabs (Beverly, MA, USA).

### 2.4. Cell Culture

Mononuclear cells were isolated by sedimentation using Ficoll-Paque Plus (GE Healthcare, Sweden) density gradient centrifugation and cryopreserved at −80°C until use. Cell pellets were resuspended in 0.5 mL of RPMI-1640 medium (Thermo Scientific HyClone), plus 10% fetal calf serum, 100 U/mL of penicillin, 100 U/mL of streptomycin, 0.5 *μ*g/mL of amphotericin B, and pH 7.0. To eliminate granulocytes, the cell pellets were frozen at −70°C and then thawed. The cells were incubated at 37°C under 5% CO_2_ and were cryopreserved in 10% DMSO.

Before performing the experiments, we prepared smears of cell suspensions using double staining with Wright and myeloperoxidase stains to confirm, by morphology, that leukemic blasts were more than 95%.

### 2.5. *In Vitro* Chemosensitivity Assay

The* in vitro *Ara-C chemosensitivity of myeloblasts was assessed with the MTT assay using a 3-day cell culture assay.

Cryopreserved cells were thawed and cultured in 96-well plates with 10,000 cells per well in triplicate. Cytarabine was added at different concentrations: 0, 0.001, 0.01, 0.1, 1.0, 10, 100, and 1000 *μ*g/mL. The control wells (Ara-C concentration 0) contained myeloblasts with culture medium, without cytarabine, and blank wells contained only medium. After incubating the cells for 72 hours at 37°C in a humidified atmosphere containing 5% CO_2_, 100 *μ*L of 10% MTT was added to each well, and the plates were incubated for additional 4 hours. Formazan crystals were dissolved by adding 100 *μ*L of isopropanol, and the optical density (OD) was measured spectrophotometrically at 570 nm. Results were considered evaluable only when the control wells contained more than 70% myeloblasts after 3 days of culture.

For every patient, cell viability was calculated for each drug concentration using the following equation (mean OD treated wells/mean OD control wells) × 100%, after correction for the background OD of the blank wells. To determine the IC_50_ value, we created *XY* data tables where *X* is log (Ara-C concentration) and *Y* is cell viability and performed a nonlinear regression analysis using the GraphPad 5 software.

### 2.6. DNA Extraction and Genotyping

Diagnostic peripheral blood samples were collected in EDTA-containing tubes. The blood was treated with ammonium chloride 1X, centrifuged at 2500 rpm for 15 minutes, washed with PBS, and centrifuged again. The cell pellet was then resuspended in 2 mL of PBS. DNA was extracted using the QIAamp DNA Blood Midi Kit (Qiagen, Düsseldorf, Germany) and stored at −20°C.

The polymorphisms C201T and C360G of the* dCK* gene and the polymorphism A719G of the* CDA* gene were identified by the PCR-RFLP (restriction fragment length polymorphism) method. Genomic DNA was amplified using a Mastercycler gradient thermocycler (Eppendorf, Germany), with oligonucleotides specific for each gene (Table 1) [21, 22].

### 2.7. Detection of the A79C Polymorphism of the* CDA* Gene

**Figure 1:**
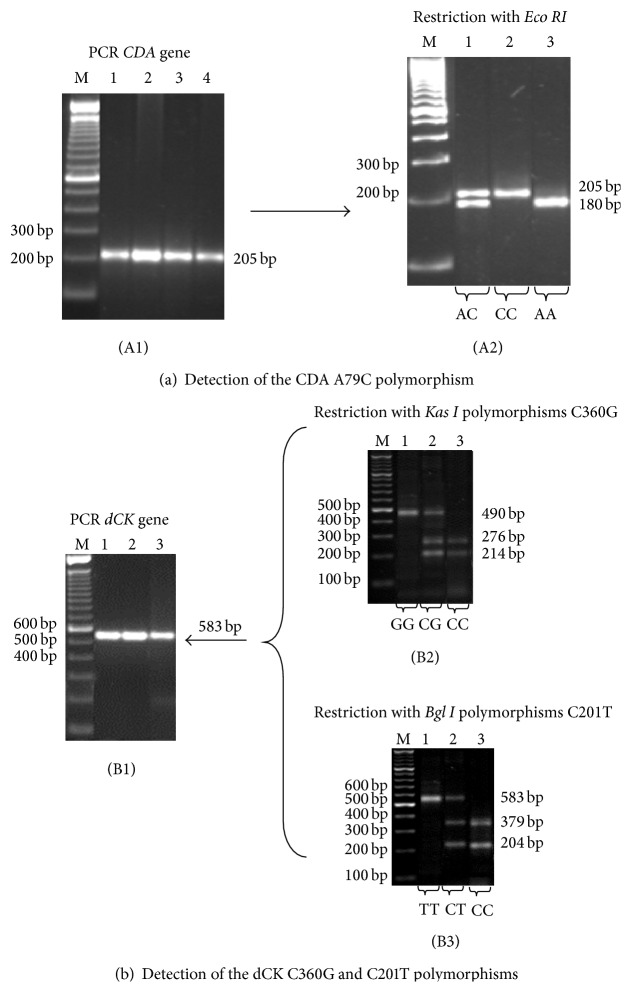
Identification of the genotypes of* CDA* and* dCK* genes. (a) Polymorphism A79C of* CDA* gene. (A1)* CDA* gene PCR. M 100 bp DNA ladder; lines 1–4: PCR product prior to digestion 205 bp. (A2) Restriction with* Eco RI*. M 100 bp DNA ladder; line 1: heterozygous (AC); line 2: variant homozygous (CC); line 3: wild-type homozygous (AA). (b) Polymorphism C360G and C201T of* dCK* gene. (B1)* dCK* gene PCR. M 100 bp DNA ladder; lines 1–3: PCR product prior to digestion 583 bp. (B2) Restriction with* Kas I*. M 100 bp DNA ladder; line 1: variant homozygous (GG); line 2: heterozygous (CG); line 3: wild-type homozygous (CC). (B3) Restriction with* Bgl I*. M 100 bp DNA ladder; line 1: variant homozygous (TT); line 2: heterozygous (CT); line 3: wild-type homozygous (CC).

Genomic DNA was amplified using oligonucleotides specific for the A79C polymorphism of the* CDA* gene, 2.5 mM dNTPs, 3 mM MgCl_2_, 1X buffer (20 mMTris-HCl and 2 mM MgSO_4_), and 0.5 U DNA Taq polymerase. The amplification conditions were as follows: initial denaturation at 95°C for 3 min followed by 40 cycles at 95°C for 40 sec, 63°C for 40 sec, and 72°C for 30 sec, with a final extension at 72°C for 1 min. A 205-bp PCR product was generated. After the amplification reaction was performed, 10 *μ*L of the PCR product was digested with 2 U of the restriction enzyme* Eco RI* at 37°C for 2 h. In the absence of the A79C polymorphism, the enzyme cuts the DNA at position 79, generating one fragment at 180 bp. When a polymorphism is present, the cutting site is eliminated and the amplified product remains the original size (205 bp) (Figure 1(a)).

### 2.8. Detection of the C201T Polymorphism of the* dCK* Gene

Genomic DNA was amplified using oligonucleotides specific for the C201T polymorphism of the* dCK* gene, 2.5 mM dNTPs, 3 mM MgCl_2_, 1X buffer (20 mM Tris-HCl and 2 mM MgSO_4_), and 0.5 U DNA Taq polymerase. The amplification conditions were as follows: initial denaturation at 95°C for 3 min followed by 40 cycles at 94°C for 60 sec, 65°C for 38 sec, and 72°C for 60 sec, with a final extension at 70°C for 5 min. A 583-bp PCR product was generated. After the amplification reaction was performed, 10 *μ*L of the PCR product was digested with 5 U of the restriction enzyme* Bgl I* at 37°C for 4 h. In the absence of the C201T polymorphism, the enzyme cuts the DNA at position 201, generating two fragments at 379 bp and 204 bp. When a polymorphism is present, the cutting site is eliminated and the amplified product remains the original size (583 bp) (Figure 1(b)).

### 2.9. Detection of the* C360G* Polymorphism of the* dCK* Gene

Genomic DNA was amplified using oligonucleotides specific for the* C360G* polymorphism of the* dCK* gene, 2.5 mM dNTPs, 3 mM MgCl_2_, 1X buffer (20 mMTris-HCl and 2 mM MgSO_4_), and 0.5 U DNA Taq polymerase. The amplification conditions and primers were the same as for the C201T polymorphism. A 583-bp PCR product was generated. After the amplification reaction was performed, 10 *μ*L of the PCR product was digested with 5 U of the restriction enzyme* Kas I* at 37°C for 4 h. In the absence of the C360G polymorphism, the enzyme cuts the DNA at position 360, thereby generating three fragments at 276 bp, 214 bp, and 93 bp. When a polymorphism is present, one cutting site is eliminated, thereby yielding two products of 490 bp and 93 bp (Figure 1(b)).

All restrictions and PCR products were visualized using UV light on 1.5% agarose gel stained with ethidium bromide.

### 2.10. Statistical Analysis

The categorical variables and* in vitro *Ara-C chemosensitivity were compared across genotypes using Fisher's exact test.

Due to the skewness of the distributions, a logarithmic transformation was performed on the chemosensitivity values. According to their chemosensitivity, the patients AML cells were classified as sensitive (IC_50_ < 2.5 *μ*g/mL), intermediate (IC_50_ 2.5–5 *μ*g/mL), and resistant (IC_50_ > 5 *μ*g/mL) [23].

Event-free survival (EFS) was calculated from the date of diagnosis until relapse or death. Overall survival (OS) was calculated from the date of diagnosis until death by any cause or last follow-up using the Kaplan-Meier method, and differences between groups were estimated by the log rank test.

The Cox model was used to identify independent prognostic variables affecting overall survival. All *P* values were considered significant when ≤0.05.

The statistical analysis was carried out with the SPSS program (version 20) and the Stata software (version 12) was used for Cox regression analysis.

### 2.11. Definitions

Complete remission was defined as a bone marrow blast count of less than 5% on morphologic examination, with absolute neutrophil count >1,000/*μ*L, platelets >80/*μ*L, and no evidence of extramedullary disease.

Nonresponders were defined as all those patients that did not achieve complete remission after three induction courses and survived the first 6 weeks of treatment.

Deaths that occurred after induction failure or relapse were classified as leukemia-related, and the deaths that were not preceded by these events were considered as treatment-related.

Early deaths were defined as the deaths that occurred within the first 6 weeks of treatment, regardless of the cause.

The IC_50_ was defined as the concentration of drug necessary for growth inhibition of 50% compared with control cells cultured in the absence of Ara-C.

## 3. Results

### 3.1. Patient Characteristics

From January 2008 to January 2013, 51 consecutive AML pediatric patients were treated in our institution; of these, 10 had PML, 5 had treatment-related AML, 3 had Down Syndrome, in 4 cases there was insufficient biomaterial for the analyses, and 2 patients refused chemotherapy. A total of 27 patients were eligible.

The median age at diagnosis was 5.91 years (ranging from 18 days to 16.5 years). Eleven (40.7%) were male. The morphological subtypes were M1 9 cases (33.3%), M2 7 (25.9), M4 8 (29.6%), M5 1 (3.7%), and M6 2 patients (7.4%).

Initial characteristics and clinical outcomes of these 27 patients are summarized in Table 2.

### 3.2. Genotype Information

The genotype frequencies of the* dCK* C201T polymorphism were C/C 9 (33.3%), C/T 11 (40.7%) and T/T 7 (25.9%) of the* dCK* C360G polymorphism were C/C 10 (37%), C/G 14 (51.9%), and G/G 3 (11.1%), and of the* CDA* A79C polymorphism were A/A 15 (55.6%), A/C 6 (22.2%) and C/C 6 (22.2%) (Table 3).

### 3.3. Therapy Response and Disease Outcomes

At the time of analysis the median follow-up was 21 months. Twenty patients (74.1%) achieved remission after the first induction course, 1 (3.7%) entered remission after the second course, 3 (11.1%) were nonresponders, and 3 (11.1%) died before remission could be assessed.

Among the 21 patients who achieved complete remission, there were 5 relapses. Two patients underwent peripheral stem-cell transplantation after relapse, one is still alive, and the other died from relapse after transplant. One patient abandoned treatment before transplant while being still in second remission, one patient developed refractory leukemia, and one was treated with chemotherapy alone; he is still alive and disease-free at 28 months after relapse.

Thirteen patients died; 8 (61.5%) were treatment-related and 5 (38.5) were leukemia-related deaths. Of them, 5 were early deaths, 4 were due to infectious complications, and one was as a consequence of pulmonary hemorrhage. The nonresponder patients died of infections while being treated with intensive chemotherapy, 3 patients died in remission during consolidation therapy, and 2 died after relapse.

### 3.4. Toxicity Analysis

Chemotherapy toxicity was recorded for a total of 115 courses of chemotherapy in 27 patients.

The 115 cycles were complicated by fever requiring intravenous antibiotic or antifungal therapy. In 24 of these events (20.8%), signs of septic shock were present and 12 patients died from septic shock.

The most common grade ≥3 toxicities observed were neutropenia in 85.2% of the cycles, thrombocytopenia in 25.2%, febrile neutropenia in 25.2%, neutropenic enterocolitis in 6%, and mucositis in 5.2%.

We found significant association between the 201 CC genotype of the* dCK *gene and hematological toxicity grades ≥3 (*P* = 0.038), and a trend towards association was found between the wild-type genotype 79AA of the* CDA* gene and hematological toxicity grades ≥3 (*P* = 0.057). No association was found between the C360G genotypes of the* dCK* gene and severe toxicity.

### 3.5. *In Vitro *Chemosensitivity

The median Ara-C IC_50_ value was 5.27 *μ*g/mL, 3 patients exhibited a sensitive pattern, 10 had intermediate sensitivity, and 14 were resistant (IC_50_ > 5 *μ*g/mL).

### 3.6. Survival Analysis

Three out of the 5 patients who relapsed were Ara-C resistant and 2 had intermediate chemosensitivity, but no significant association could be demonstrated between in* vitro *sensitivity to Ara-C and the risk of relapse (*P* > 0.1).

We found a trend toward association between the wild-type genotype 360 CC of the* dCK* gene and* in vitro* resistance to Ara-C (76.9% of the wild types were resistant versus 35% of the variants) (*P* = 0.056). And the same was found with the wild-type genotype 201 CC (81.8% of the wild types were resistant versus 36.4% of the variants) (*P* = 0.064). There was no association between the A79C polymorphisms and* in vitro *Ara-C chemosensitivity.

**Figure 2:**
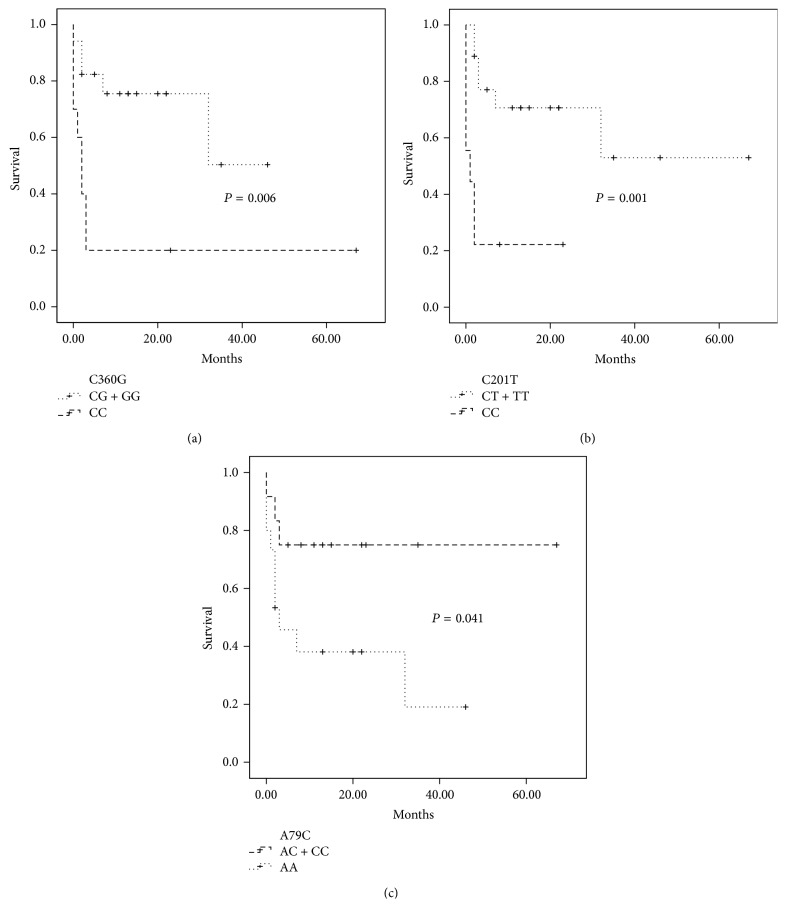
Kaplan-Meier curves of overall survival by genotypes. (a)* dCK* C360G SNPs, (b)* dCK* C201T SNPs, and (c)* CDA* A79C SNPs.

**Figure 3:**
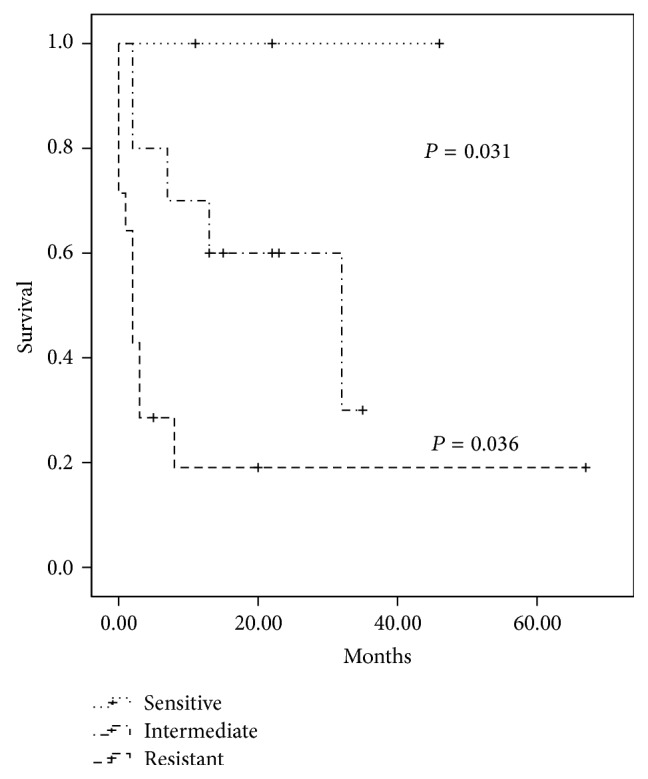
Kaplan-Meier curve of overall survival stratified according to the* in vitro* Ara-C sensitivity.

The survival analysis using the Kaplan-Meier method revealed that, in the C360G polymorphisms of the* dCK* gene, the variant genotype significantly increased OS (50.3% versus 20%) (*P* = 0.006) and EFS (37.8% versus 20.0%) (*P* = 0.028). For the C201T polymorphism of the* dCK* gene, the outcome was significantly worse for the patients carrying the wild-type genotype OS (53% versus 22.2%) (*P* = 0.001) and EFS (44.7 versus 11.1%) (*P* = 0.001). For the A79C genotypes of the* CDA* gene, we observed a significant difference in OS (75% versus 38.1%) (*P* = 0.041) and a marginal difference in EFS among patients who carried the variants 79AC and 79CC compared to those with the wild type (Figure 2). We also found a significant difference between the* in vitro* Ara-C sensitivity pattern and survival (Figure 3). The patients who exhibited a resistant pattern (*n* = 14) had a significantly worse OS (19%) compared to the patients that had an intermediate sensitivity (30%) (*P* = 0.036) and to those who were sensitive to Ara-C (100%) (*P* = 0.031), while age, gender, subtype, and leukocyte counts at diagnosis did not have an impact on survival.

### 3.7. Cox Regression Analysis

In the multivariable Cox regression analysis, we included age, gender, FAB subtype, leukocyte count at diagnosis, response to the first induction course,* in vitro *chemosensitivity, C360G SNPs and C201T SNPs of the* dCK* gene, and A79C SNPs of the* CDA *gene. We found that the most significant prognostic factors were age at diagnosis, wild-type genotype of the A79C polymorphism, and wild-type genotype of the C360G polymorphism (Table 4). Gender, FAB subtype, leukocyte counts at diagnosis, response to the first induction course, and Ara-C* in vitro *chemosensitivity did not show significant effect on the risk of death.

## 4. Discussion

Treatment of acute myeloid leukemia remains a challenge worldwide. It is a fact that lower-income countries have not achieved the same survival rates as high-income countries, and their results are not easily reproducible despite the use of the same protocols [24].

In low-income countries, treatment-related mortality remains a significant cause of treatment failure, while in high-income countries relapse is the leading cause of death. Possible explanations for this difference include variations in supportive care interventions, sociocultural aspects, nutritional status, and genetic factors.

Cytarabine is the essential drug in AML induction and consolidation chemotherapy. The response to and the toxicity of Ara-C treatment are characterized by significant interindividual differences, and the development of Ara-C resistance is still a major problem, raising the possibility that the pharmacogenetics of this agent may play a role in the difference of outcomes. Many single nucleotide polymorphisms (SNPs) and haplotypes of* dCK* and* CDA*, which contribute to susceptibility to Ara-C, have been identified in Africans, Europeans, and Chinese [24–26]; however, there are few reports about the relation between dCK and CDA polymorphisms and AML outcome [18–20].

We analyzed the impact of the polymorphisms of genes encoding cytidine deaminase (CDA A79C) and deoxycytidine kinase (dCK C360G, C201CT) and of* in vitro* sensitivity to Ara-C on disease outcome and toxicity in 27* de novo* pediatric AML patients.

In this study, treatment-related mortality represented the main cause of failure (61.5% of the deaths), similar to the mortality pattern reported in other Latin American countries [24].

We found that children with the 79CC genotype of the* CDA* gene and the 360CC genotype of the* dCK* gene had significantly higher risk of dying, and the 201CC genotype was also associated with higher incidence of hematological toxicity grades ≥3, as well as less probability of survival.

In a previous study, Children's Oncology Group demonstrated that children with a low-activity 79CC genotype were at an increased risk of treatment-related mortality [19]. Some* in vitro* studies have shown that increased CDA enzyme activity has a role in the development of resistance to cytarabine [27–29]. The A79C polymorphism has also been associated with Ara-C toxicity [30], while the dCK-360G allele was found to increase the risk of mucositis after exposure to low-dose cytarabine in childhood ALL therapy [31].

Shi et al. reported an association between genotypes 360CC and 201CC of the* dCK* gene and poor clinical outcome in AML patients treated with Ara-C-based chemotherapy [25]. These authors demonstrated that the transcriptional activity of the 5′ flanking region of the homozygous variants of the* dCK* gene was eightfold greater than that of the wild type, suggesting that variant genotypes may provide acellular transcriptional machinery with more efficient promoter/enhancement elements.

Age was one of the most significant prognostic factors, as has been found by previous pediatric studies [32, 33]. The Nordic Society for Pediatric Hematology and Oncology reported that children of 10 years of age or older had an inferior prognosis compared to younger children [3].

In our study,* in vitro* resistance to Ara-C seemed to be associated with low probability of survival, although this did not achieve statistical significance in multivariate analysis, possibly due to the limited sample size.

We were not able to demonstrate an association between response to the first induction course and disease outcome, as has been previously described by the NOPHO and other groups. This is probably related to the fact that most of the patients died relatively early from causes related to treatment and not as a result of resistance.

Early identification of patients at risk of severe toxicity allows for early intervention to reduce morbidity and mortality; equally important is the identification of those cases at risk of developing chemoresistance. Regarding the pharmacogenomics of cytarabine, clinical studies have identified a biomarker (Ara-CTP levels) and a main candidate gene (*dCK*), while cell-based models have identified candidate SNPs associated with these phenotypes.

We tested the hypothesis that the analysis of drug targets for polymorphism can help to establish gene-based information for the treatment of AML patients; we investigated functional SNPs in the* CDA* and* dCK* genes and found that some polymorphisms have a role in predicting survival and toxicity.

In a hospital like ours, where intensive care support, transfusion therapy, and appropriate treatment of infections are not a major problem, such a high rate of treatment-related mortality would not be expected. Thus, we believe that genetic factors are contributing to a higher frequency of serious adverse events leading to lower survival rates.

The frequency of homozygous wild-type genotype 79 AA of the* CDA* gene observed in this study was higher than that found in Caucasian populations [19, 34–36], who report the highest rates of survival in AML; however, it was lower than that of Asian populations [18, 37], suggesting that this genotype might be more common in Mexican than in Caucasian populations. Regarding the frequency of the wild-type genotype 360CC of the* dCK* gene, we found a lower frequency than that described in Chinese and French populations [25, 34], although there are few reports about the frequencies of these polymorphisms. These results must be interpreted with caution because they are based on a small number of patients and, although we found that both genotypes represent a risk factor, there are several enzymes involved in the metabolic pathway of Ara-C and various polymorphisms can be present in each. Further prospective studies on the clinical impact of these SNPs are warranted in large cohorts of Mexican/Hispanic ad in other populations to verify these results.

Pharmacogenetics has become a major concern in personalized medicine; some markers such as TPMT, DPYD, or UGT1A1 are routinely used in clinical practice. The next challenge will be to incorporate new genotype-phenotype correlations into clinically useful diagnostics and prognostic stratification.

## 5. Conclusion

Despite the limited sample size, some of our results are statistically significant and consistent with previous reports. We consider that these findings add evidence to the role that polymorphisms C360T and C201T of the* dCK *gene and A79C of the* CDA* gene have to anticipate toxicities and predict treatment outcome and toxicity in patients with AML treated with cytarabine-based chemotherapy.

## Figures and Tables

**Table 1:** Primer sequences used for amplification of the *dCK* and *CDA* genes.

Gene	Sequence	Product (bp)
*dCK A *	5′ GCC TTC TCC CCA GAT GAG TT 3′	583 bp
*dCK B *	3′ CAC TGG CGG GCC TGC GGG 5′	
*CDA A *	5′ GAC ACA CCC AAG GGG AGG A 3′	205 bp
*CDA B *	3′ GAC TGT AGG GGC AGT AGG CTG AAT 5′	

**Table 2:** Initial characteristics and clinical outcomes of 27 pediatric patients with AML.

Number of patients	27
Age, median (yr), range	5.9 (0.05–16.5)
Sex	
Male	11 (40.74)
Female	16 (59.26)
Total blood counts	
Leucocytes, median (range)/uL	23,000 (3,100–254,000)
Platelets, median (range)/uL	36,000 (3,000–152,000)
Hemoglobin, median (range) g/dL	6.8 (2.8–13.0)
WBC, >100,000/uL, *n* (%)	8 (29.6)
CNS positive, *n* (%)	2 (7.4)
FAB, *n* (%)	
M1	9 (33.3)
M2	7 (25.9)
M4	8 (29.6)
M5	1 (3.7)
M6	2 (7.4)
M7	0
Remission	
After the first induction course *n* (%)	20 (74.1)
After the second induction course *n* (%)	1 (3.7)
Nonresponders *n* (%)	3 (11.1)
Not assessed	3 (11.1)
Relapses *n* (%)	5 (18.5)
Deaths *n* (%)	13 (48.1)
Early deaths	5/13 (38.4)
Treatment-related	8 (61.5)
Leukemia-related	5 (38.5)

**Table 3:** Frequencies of C201T and C360G polymorphisms of the *dCK* gene and A79C of the *CDA* gene in 27 Mexican pediatric patients with acute myeloid leukaemia.

*CDA* gene		*dCK* gene			
Polymorphism A79C		Polymorphism C360G		Polymorphism C201T	
Genotype	Frequency % (*n* = 27)	Genotype	Frequency % (*n* = 27)	Genotype	Frequency % (*n* = 27)
AA	15 (55.6%),	CC	10 (37%)	CC	9 (33.3%)
AC	6 (22.2%)	CG	14 (51.9%)	CT	11 (40.7%)
CC	6 (22.2%).	GG	3 (11.1%)	TT	7 (25.9%)

**Table 4:** Multivariate analysis (Cox proportional hazards model) of overall survival in patients with AML treated with Ara-C (*n* = 27).

Regressors	Hazard ratio	[95% Conf. Interval]
Age at diagnosis	0.817	0.683–0.979
Wild-type 79AA	8.314	1.798–38.436
Wild-type 360CC	11.064	1.588–77.056
Wild-type 201CC	0.769	0.140–4.231
